# Transactions of the Indiana State Medical Society, at Its Seventh Annual Session

**Published:** 1857-01

**Authors:** 


ART. VII.—*Transactions of the Indiana State Medical Society*, at its Seventh Annual Session, held in the City of Indianapolis, May 20, 1856.

THE work before us is an octavo pamphlet of seventy-five pages, and contains a number of very valuable reports. The paper to which our attention has been more particularly directed, is the "Report of Dr. J. S. Bobbs on Surgery." Among the cases detailed in this report is one of melanotic tumor of the orbit, occurring in the practice of Dr. Bobbs. The history of this case is an exceedingly interesting one, and is accompanied by a remarkably life-like lithograph of the patient's face, with the diseased mass projecting from the orbit. The tumor was excised by Dr. Bobbs, and at the date of the report, two years and a half after the operation, there had been no return of the disease. Another instructive case embraced in the report is one of "Compound Dislocation of the Carpus," the history of which was furnished by Dr. M. H. Harding, of Lawrenceburg; and as it seems to illustrate a practice which is sometimes necessary in such injuries, we copy it in full:

"In the March number of the London *Lancet*, Samuel Solley, Esq., Surgeon to St. Thomas's Hospital, reports a case of compound dislocation of the forearm at the carpus, in which excision of the lower end of the radius was necessary to effect reduction, and urges attention to the adoption of such means as are necessary to prevent anchylosis of the joint by adhesion of the tendons to the new osseous deposition.

"As such injuries are rare, and some discrepancy of opinion exists as to the proper treatment, even among the highest authorities in the profession, and there having occurred in my practice, some years since, a case of almost precisely similar character, and resulting from the same cause, I have thought that a report of it might be of some interest in eliciting the opinion of the profession as to the proper treatment in such cases. The following report is taken from notes made at the time of the injury. On August 4th, 1839, I was called to see P. B., aged 14 years, who had fallen from a tree at a height of about 18 feet from the ground. His elder brother, who was with him and saw him fall, says that he fell head foremost with the arms extended, but that the principal force of the fall was received upon the right extended hand and arm. The violence of the fall produced a compound dislocation of the radius and ulna, throwing the hand backward upon the posterior face of the forearm, and necessarily lacerating and breaking away most of the soft parts on the anterior part of the wrist-joint. The radial and ulnar arteries were, however, uninjured. The bones of the forearm, by the force of the fall, were driven several inches into the ground, as was indicated by the debris of decaying leaves and abundant earth with which their carpal extremities were covered. I saw the patient about four hours after the receipt of the injury. I found the hand and lacerated muscles much swollen and retracted; the radius protruding through the lacerated soft parts on the anterior face of the hand just below the wrist-joint. Time enough had elapsed since the receipt of the injury, for reaction to be pretty well established from the effects of the concussion, and the shock to the nervous system incident to so violent and extensive an injury of the soft parts. He was collected in mind, and not suffering as much pain as might have been anticipated from the character and extent of the injury. After making suitable preparations, assisted by two men, I endeavored to reduce the dislocation by forcibly flexing the hand, and drawing down in direction of the axis of the forearm, the arm and forearm being fixed. And although assisted by two strong men, I could not succeed in doing so by any force that I thought safe to apply, without incurring the risk of more serious injury to the already weakened and lacerated structures surrounding the wrist-joint. Fearing more serious injury to the joint by any further effort at reduction, I determined to remove the end of the radius by means of the metacarpal saw. I had visited the case without knowing the character of the injury, and at this juncture found myself four miles from my office, and under the necessity of returning to it for my instruments, before I could proceed with the operation. I enveloped the injured part in cloths kept constantly wet in cold water, and went to my office for necessary instruments with which to excise the bone, and returned about six hours after the receipt of the injury, removed three-fourths of an inch of the carpal extremity of the radius, and effected reduction without difficulty. My recollection is, that the periosteum was not separated at the point of excision, although there is no mention of the fact in the notes referred to. The hand and forearm were placed on a well-padded splint in a supine position. This was secured by a roller bandage, leaving the wound on the front of the forearm bare, the limb placed on a pillow, and cold water dressings applied to the wound.

"August 6th. Patient has rested pretty well since the reduction of the dislocation. Hand and forearm very much swollen, but pain not very acute; pulse 115 per minute; bowels not having been moved for the last 48 hours; ordered 10 grs. sub. mu. hydrarg., succeeded by dose ol. ricini, if the bowels are not freely moved; wound looks healthy, and beginning to suppurate; continue the cold water dressings to hand and arm, and ung. resinæ to wound.

"August 9th. Case progressed favorably since seen last; swelling and constitutional disturbance passing away; wound granulating and closing up finely; continue dressing on the wound.

"August 15th. Made the last professional visit to-day; wound nearly closed; directed gentle flexion and extension of the fingers, with alternate pronation and supination of the hand. In eight weeks from the receipt of the injury, the patient had recovered very perfect use and control of hand and forearm. I have frequently seen this patient, years after the receipt of the injury, and I could not see that he suffered either deformity or disability to any appreciable extent in the injured limb.

"The only point of great practical importance, in either the case reported by Mr. Solley or myself, is, the little importance that either of us gave to the necessity of removing the bone at as high a point as there was separation of the periosteum, without any regard to the amount positively necessary to effect a reduction of the dislocation. I think that point was reached in the case above reported, and hence its speedy and favorable issue without an untoward symptom; although I am candid enough to admit that result may have been reached rather by accident than design, as surgical authorities, so far as I know, never have directed especial attention to this important consideration in the successful treatment of compound dislocations, when excision of the protruding bone is necessary; in my own case, only having regard to the necessity of removing so much as would enable me to effect reduction without too much violence to the soft parts. The report of Mr. Solley's case illustrates the importance of the practice in an eminent degree. He saw his case on the 20th of June, and removed one-eighth of an inch of the radius, which he says was denuded of periosteum, and reduction was readily effected. The case was then under daily treatment until the 1st of November, at which time, the wound not having healed, necrosis was suspected, and found, on examination, to exist, and one inch and a half of the necrosed extremity of the radius was removed, and against the 1st of January, six months and a half from the receipt of the injury, the wound was closed with a fair prospect of having an unimpaired hand and arm. Mr. Solley further remarks, in detailing the treatment of his case, 'I have no doubt that the periosteum had been stripped off at the time of the accident, or rather that the bone was detached from the periosteum, which being left in the arm,' he continues,

'will generate new bone, and a useful arm will be obtained,' without, however, alluding to what would have been better practice, to have removed the bone rather in reference to the extent of the separation of the periosteum, than the amount necessary to reduction of the dislocation. Another point of some practical importance grows out of a remark quoted above, 'the periosteum being left, new bone will be re-produced.' Is the periosteum the only source of ossific deposit? And to what extent may solution of continuity of the ossific structure be ventured upon with a prospect of re-deposition?"

The next two papers are a short "Abstract of Reports presented to the Cambridge City Medical Association for the year ending April 1, 1856, by Dr. L. R. Johnson," and a "Report on Microscopic Pathology, by Dr. David Hutchison, of Mooresville." Succeeding these are two reports on Erysipelas, one by Dr. George Sutton, of Aurora, and the other by L. Ritter, of Plainfield. We extract Dr. Sutton's brief notice of an epidemic of this formidable disease; and would call attention to the question concerning its connection with other diseases which prevailed at the same time:

"The epidemic form prevailed in the southeastern part of Indiana, in the counties of Dearborn, Ripley, and Decatur, in the years 1842 and 1843; and it is a fact worthy of attention, that it did not appear simultaneously in these counties, but spread over the country as erysipelas spreads over the body, passing gradually from one point to another. In November, it was prevailing near Napoleon, in Ripley County, in a very malignant form; by the first of January, it had reached Moors Hill, a small town some ten or twelve miles in a northeasterly direction; spreading on in the same direction, the disease extended over a belt of country reaching from Hartford to Manchester, about ten miles in breadth. It soon made its appearance in Wilmington in the same direction, and the practising physician of that place being severely attacked with the disease, the whole practice of that town and neighborhood came under my care; giving me an opportunity of seeing this disease in almost every form, before it reached Aurora. In May, it made its appearance in the neighborhood of Aurora, and it was the principal disease we had until November. Late in the fall it reached Lawrenceburg, and prevailed there during the winter; at the same time it made its appearance across the river in Boone County, Ky., and prevailed in the neighborhood of Bellevue. During its prevalence in the neighborhood of Aurora, Wilmington, and Manchester, a variety of diseases appeared simultaneously with it. We had puerperal fever, various forms of pneumonia, cynanche trachealis, tonsillitis, laryngitis, gastro-enteritis, phlebitis, and diffuse inflammation of every variety. These diseases increased in frequency as the erysipelas became more prevalent, and declined as it subsided. The question soon arose, are all these different diseases, modified by the prevailing epidemic, or are most of them forms of internal erysipelas? From a similarity of constitutional symptoms, and the intimate connection of erysipelas with many of these diseases, I was led to adopt the latter view; most of our books taught us, however, that erysipelas was a disease of the skin alone, and we find this view still advocated in many of our best medical works. But facts have been continually coming under my observation, which sustain the view just taken, and irresistibly impel me to regard those authors as correct, who consider erysipelas as a form of inflammation, not confined to the skin, but capable of spreading along other tissues. We want the united observation of physicians on this subject.

"That erysipelas occasionally attacks the mucous membranes, I think can be demonstrated to the senses. I have seen erysipelas spread in a well-circumscribed line over the roof of the mouth to the inside of the cheek and angle of the upper lip, and from thence over the face and head."

The remaining "Reports" are one on Stomatitis Materna, by Dr. J. S. McClelland, of Jefferson; on Diseases of the Eye, by Dr. Daniel Meeker, of Laporte; on Vital Statistics, by Dr. W. Lomax, of Marion; and an Address on European Hospitals, by Dr. T. J. Cogley, of Madison.

